# Usefulness and Limitations of PFGE Diagnosis and Nucleotide Sequencing Method in the Analysis of Food Poisoning Pathogens Found in Cooking Employees

**DOI:** 10.3390/ijms25074123

**Published:** 2024-04-08

**Authors:** Mi-Na Park, Sang-Gu Yeo, Junhyuk Park, Yoomi Jung, Se-Min Hwang

**Affiliations:** 1 Graduate School of Public Health & Welfare, Konyang University, 158 Gwanjeodong-ro, Daejeon 35365, Republic of Korea; enne4638@naver.com; 2 Chungcheongnam-do Institute of Health and Environment Research, 8 Hongyegongwon-ro, Hongseong 32254, Republic of Korea; junhyuk@korea.kr; 3 Korea Disease Control and Prevention Agency, Osong Health Technology Administration Complex, 2 Osongsaengmyeong-ro, Cheongju 28159, Republic of Korea; yeosg@korea.kr; 4 Korea Armed Forces Nursing Academy, 90 Jaun-ro, Daejeon 34059, Republic of Korea; ymjungbest@korea.kr; 5 Department of Preventive Medicine, Myunggok Medical Faculty, Medical Campus, Konyang University College of Medicine, 158 Gwanjeodong-ro, Daejeon 35365, Republic of Korea; 6 Myunggok Medical Research Center, Konyang University College of Medicine, 158 Gwanjeodong-ro, Daejeon 35365, Republic of Korea

**Keywords:** food poisoning, outbreak, PFGE, nucleotide sequencing, *S. aureus*, *EPEC*, Norovirus

## Abstract

In the case of a food poisoning outbreak, it is essential to understand the relationship between cooking workers and food poisoning. Many biological diagnostic methods have recently been developed to detect food poisoning pathogens. Among these diagnostic tools, this study presents PCR-based pulsed-field gel electrophoresis and nucleotide sequencing diagnostic analysis results for diagnosing food poisoning outbreaks associated with cooking employees in Chungcheongnam-do, Republic of Korea. Pulsed-field gel electrophoresis was useful in identifying the food poisoning outbreaks caused by *Staphylococcus aureus* and *Enteropathogenic Escherichia coli.* In the case of Norovirus, nucleotide sequencing was used to identify the relationship between cooking workers and the food poisoning outbreak. However, it is difficult to determine whether cooking employees directly caused the food poisoning outbreaks based on these molecular biological diagnostic results alone. A system is needed to integrate epidemiological and diagnostic information to identify a direct correlation between the food poisoning outbreak and cooking employees.

## 1. Introduction

Foodborne disease (FBD), also referred to as foodborne illness or food poisoning, is caused by consuming contaminated food or drink [1]. It can be caused by a variety of different bacteria (*Bacillus cereus*, *Campylobacter* spp., *Clostridium botulinum*, *Clostridium perfringens*, *Cronobacter sakazakii*, *Esherichia coli*, *Listeria monocytogenes, Salmonella* spp., *Shigella* spp., *Staphylococccus aureus*, *Vibrio* spp., and *Yersinia enterocolitica*), viruses (Hepatitis A and Noroviruses), and parasites (*Cyclospora cayetanensis*, *Toxoplasma gondii* and *Trichinella spiralis*), and such pathogens have been reported as linked to some important outbreaks [2]. The WHO estimated that foodborne diarrheal diseases caused 600 million cases and 420,000 deaths worldwide a year [3].

In particular, when there is a cooking staff member who has food poisoning symptoms but is not excluded from the job, the incidence rate of patients with the disease increases by as high as 226% [4,5]. Despite this, previous studies reported that as many as 60% of food employees had worked while ill and 20% while experiencing vomiting or diarrhea [4,6,7]. Food employees cited a loss of pay, light symptoms, and the workload of remaining staff as the influencing factors which led them to staying in work [6,7,8]. The typical example of a cook-caused food poisoning epidemic is Mary Mallon [9], also known as “Typhoid Mary” (1869–1938). She was a healthy carrier of *Salmonella typhi* with no symptoms and worked as a cook in different households and restaurants in several areas in the United States. As a result, at least 122 people were infected, and five of them died due to the disease. This case demonstrates that it is essential to quickly detect food poisoning in cooking employees and exclude them from work until at least 48 h after symptoms have been cured [4].

Recently, many diagnostic methods have been developed to detect food poisoning. Such methods include the use of culture-based methods, immunological assays, nucleic acid-based methods, polymerase chain reactions (PCR), next-generation sequencing (NGS) methods, and biosensors [10,11,12].

This study presents PCR-based pulsed-field-gel electrophoresis (PFGE) and nucleotide sequencing diagnostic analysis results for diagnosing the food poisoning outbreak associated with cooking employees in Chungcheongnam-do, Republic of Korea. It shows the usefulness and limitations of the molecular biological diagnostic methods. Based on the results, we attempt to suggest a more effective way to diagnose the cause of food poisoning and to conduct an epidemiological investigation of food poisoning related to cooking employees.

## 2. Results

### 2.1. PFGE Results of the Types of Bacteria Isolated in the Food Poisoning Outbreak

**Figure 1:**
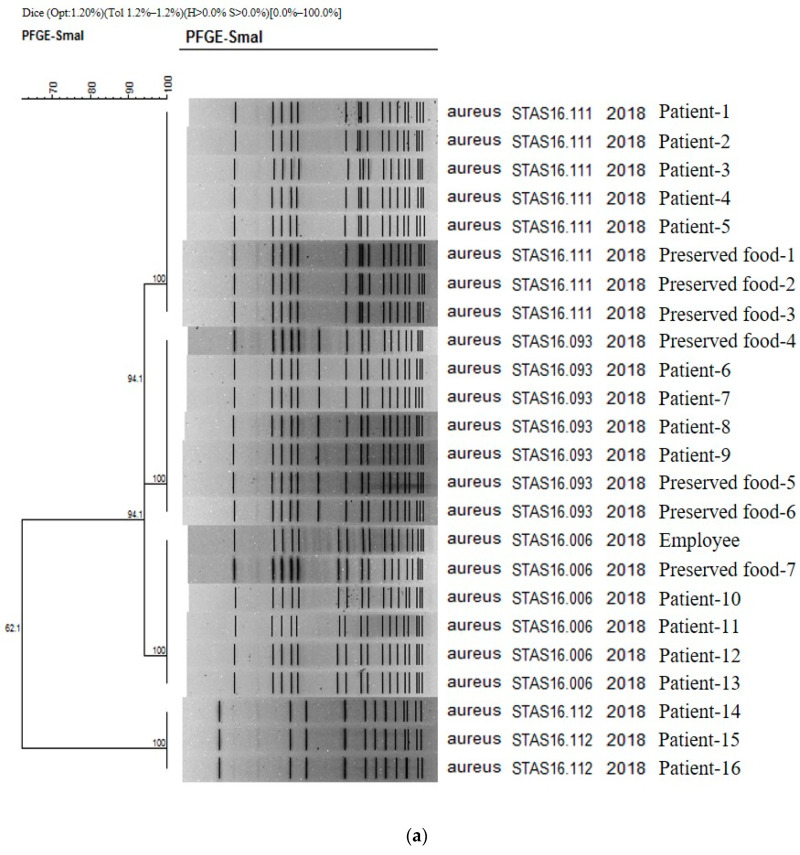

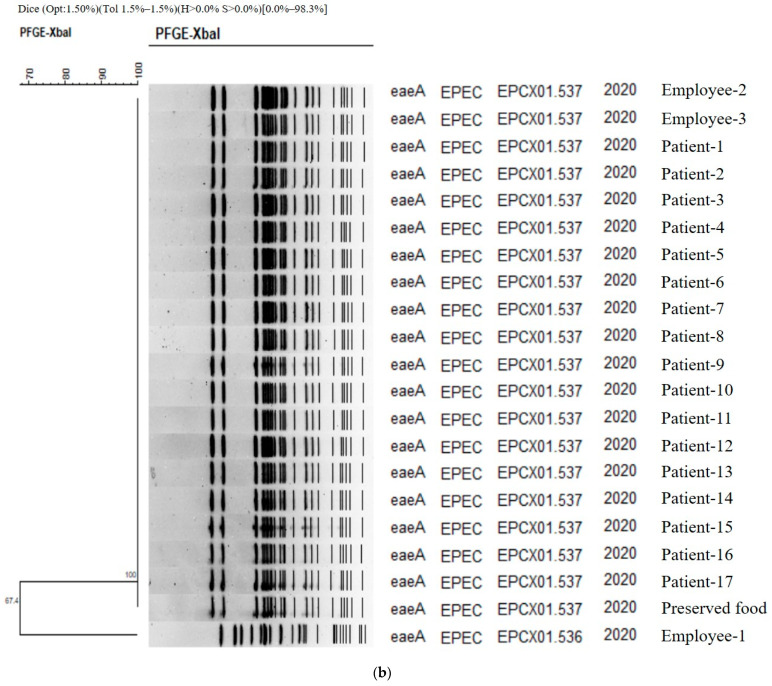
(**a**) PFGE showing clustering of the types of bacteria isolated in the food poisoning outbreak in Chungcheongnam-do, Republic of Korea: STAS type of the *S. aureus* from Outbreak 1. (**b**) PFGE showing clustering of the types of bacteria isolated in the food poisoning outbreak in Chungcheongnam-do, Republic of Korea: EPCX type of the EPEC from Outbreak 4.

The results obtained in PFGE study confirmed the presence on 16, 1, and 7 strains of Staphylococcus aureus isolated from patients, cooking employees, and preserved foods, respectively (Figure 1a). As a result of PFGE analysis of 21 stocks related to *S. aureus* from Boryeong food poisoning, four PFGE types were confirmed: STAS16.111 (8 strains), STAS16.093 (7 strains), STAS16.006 (6 strains), and STAS16.112 (3 strains). It was found that four different types of *S. aureus* acted simultaneously in Outbreak 1. The similarity between each type ranges from 94.12% to 62.07%. As a result of checking the PulseNetKorea DB, a domestic PFGE database, STAS16.111, and STAS216.112 were the first cases identified in Republic of Korea. The PFGE types of the 16 analyzed patients were evenly distributed as STAS16.111 (5 strains), STAS16.093 (4 strains), STAS16.006 (4 strains), and STAS16.112 (3 strains). For the cooking employees, the pathogen was confirmed as STAS16.006, and for the preserved food, it was confirmed as STAS16.111 (3 strains), STAS16.093 (3 strains), and STAS16.006 (1 strain) (Figure 1a).

In Outbreak 4, food poisoning bacteria and viruses were tested on 82 patients, 16 cooking employees, 135 preserved foods, and 12 environmental samples, including cooking utensils, such as cooking water, knives, and cutting boards: EPEC was detected in 12 patients, three cooking employees, one preserved food, and 13 patients, and *C. perfringens* was detected in the same samples as well. As a result, EPEC was detected in one sample out of 135 preserved foods and not in any other environmental samples (Table 1).

The authors carried out a genetic homology analysis to analyze the relationship between EPEC strains isolated from patients, food employees, and preserved foods. PFGE was performed for 17, 3, and 1 strain, respectively. As a result, two types were identified (Figure 1b). Except for one cooking employee, Employee 1, (EPCX01.536), the genetic fingerprint patterns of all strains (EPCX01.537) isolated from one case of preserved food, two cooking employees, and 17 patients were 100% identical. The homology between Employee 1 and the other two was 67.4%, and these two genetic patterns—EPCX01.536 and EPCX01.537—were the first types identified in the Republic of Korea according to PulseNetKorea DB (Figure 1b). As a result, the EPEC strain isolated from Employee 1 was concluded to be unrelated to this food poisoning.

In the case of C. perfringens and EAEC, PFGE was not performed because they did not fall under the PFGE target bacteria suggested by the Korea Disease Control and Prevention Agency.

### 2.2. Nucleotide Sequencing Results of the Types of Norovirus Isolated in the Food Poisoning Outbreak

The obtained results from the test on food poisoning bacteria or viruses carried out on 61 people confirmed that the prevalence or detection of Norovirus GII was 22/29 (75.9%), and Norovirus GI and Norovirus GII were detected in 12/32 (37.5%) cooking employees. None were detected in the bacterial test. Seven water and environmental samples were tested, including four groundwater samples, two knives, and one cutting board. Among others, Norovirus GII was detected in two of the four groundwater samples, and the same virus was found in the samples of the patients and the cooking employees as well in Outbreak 2 (Table 1). 

The base sequence was analyzed to determine the genotype of the Norovirus. The detailed genotypes were GII.2 type in 1 patient, GII.3 type in 9 patients, and GII.17 type in 12 patients. Among 12 cases, including one superinfection in the cooking employees, 1 case was GI.7 type. There were three cases of type GI.8, five cases of type GII.3, one case of type GII.8, and three cases of type GII.17. The two groundwater samples were types GII.3 and GII.8, respectively.

**Figure 2:**
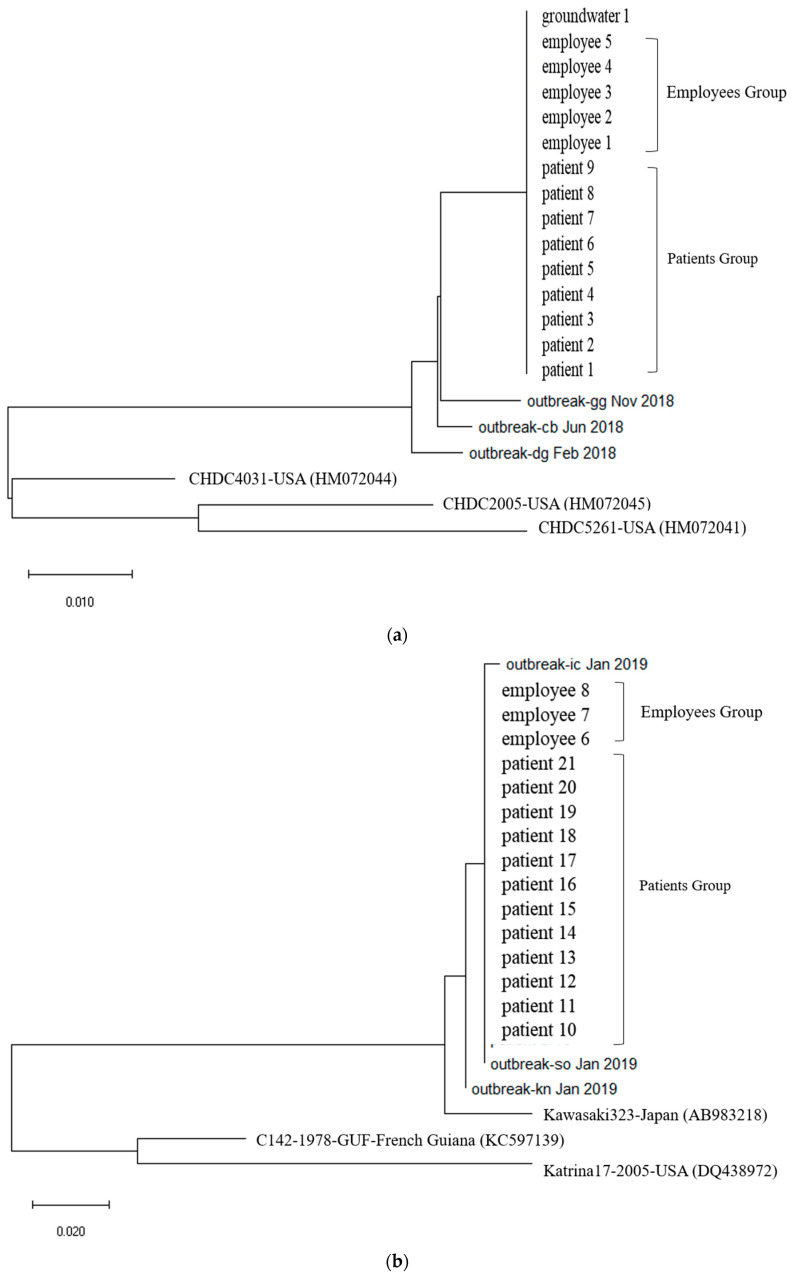
(**a**). Phylogenetic tree showing clustering of Norovirus strains isolated in food poisoning outbreak in Chungcheongnam-do, Republic of Korea: GII.3 strains of the Norovirus from Outbreak 2. (**b**) Phylogenetic tree showing clustering of Norovirus strains isolated in food poisoning outbreak in Chungcheongnam-do, Republic of Korea: GII.17 strains of the Norovirus from Outbreak 2.

**Figure 3:**
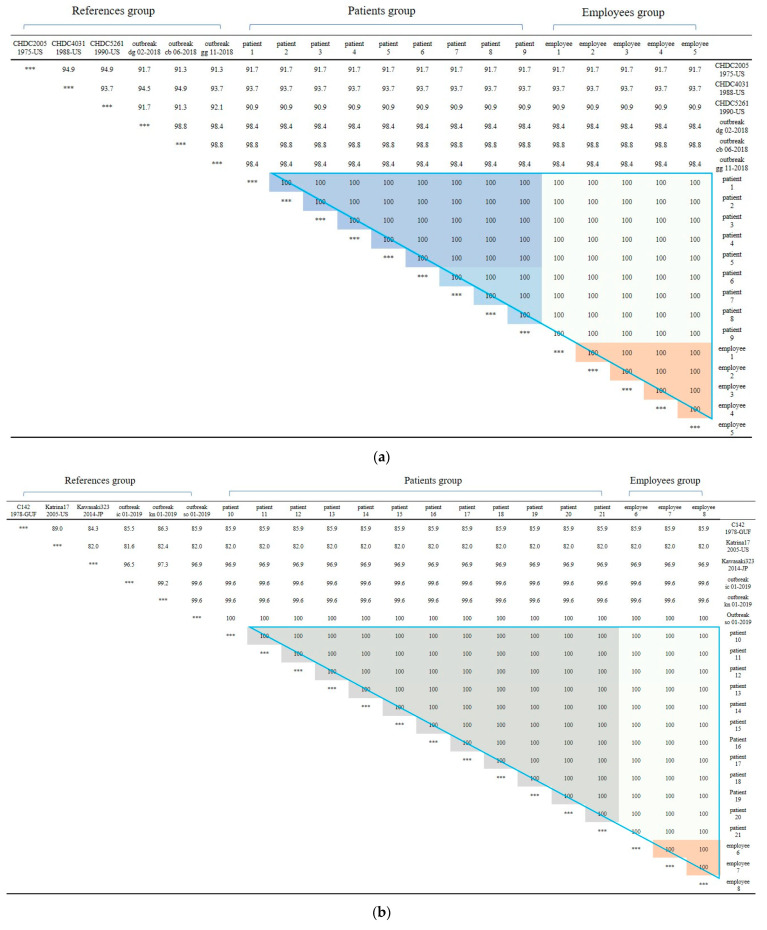
(**a**) Nucleotide sequences showing clustering of Norovirus strains isolated in food poisoning outbreak in Chungcheongnam-do, Republic of Korea: GII.3 strains of the Norovirus from Outbreak 2. (**b**) Nucleotide sequences showing clustering of Norovirus strains isolated in food poisoning outbreak in Chungcheongnam-do, Republic of Korea: GII.17 strains of the Norovirus from Outbreak 2. *** It meant that the variables on the X-axis and the Y-axis were the same. Blue big triangle meant patients and employees clustering of GII.17 strains of the Norovirus; the gray color meant patients clustering; the light gray color meant employees between patients clustering; and the orange color meant employees clustering.

First, In Outbreak 2, as GII.3 and GII.17 was the virus type which was most frequently detected in the patients, multiple alignment was performed on them, and phylogenetic analysis was requested based on this. In the case of the isolated Norovirus type GII.3, it was detected in patients, cooking employees, and groundwater. As a result of analysis, the detected Noroviruses were 100% identical in the capsid region gene sequence of 254 bp, confirming that the Norovirus that led to the epidemic was the same isolate. It showed 98.4% to 98.8% homology with Norovirus type GII.3, which caused an outbreak in other parts of the country in 2018, and 90.9 to 93.7% homology with isolates from the United States (Figure 2a and Figure 3a).

The isolated Norovirus GII.17 type was detected in 12 patient and 3 cooking employee. As a result of analysis, the capsid portion gene sequence of the detected Norovirus was 100% identical at 255 bp, confirming that the Noroviruses detected in the patient and cooking employee were the same isolate. This isolate showed 99.6% to 100% homology with Norovirus type GII.17, which caused an outbreak in other parts of the country in 2019, and 96.9% homology with “Katrina17” from the United States in 2005 (Figure 2b and Figure 3b).

**Figure 4:**
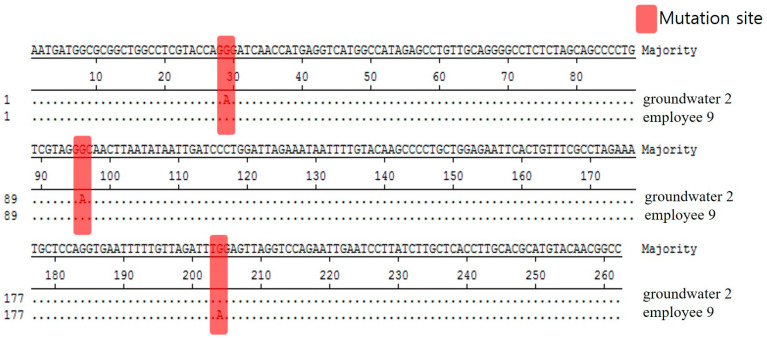
Comparison of nucleotide sequences for Norovirus GII.8 strains isolated in food employees and groundwater from Outbreak 2.

We analyzed and compared the 262 bp base sequence to see whether Norovirus GII.8, detected in groundwater and employee among the Noroviruses detected in this food poisoned group, is the same. Based on the result that the base sequences were analyzed differently at three locations, it was determined that these two isolates were different (Figure 4).

Food poisoning bacteria and viruses were tested on 45 people (39 patients and 6 cooking employees) in Outbreak 3: EAEC was detected in 21 of 39 patients, *S. aureus* in 7, and Norovirus in 17. There were coinfection cases: three patients with EAEC and *S. aureus*, four with Norovirus and *S. aureus*, and nine with Norovirus and EAEC. EAEC was detected in one out of six cooking employees (Table 1).

**Figure 5:**
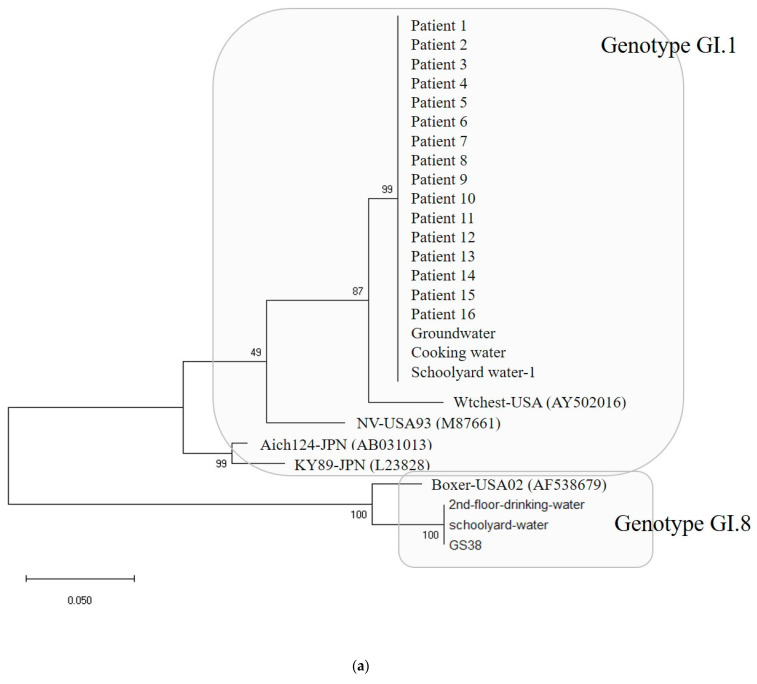

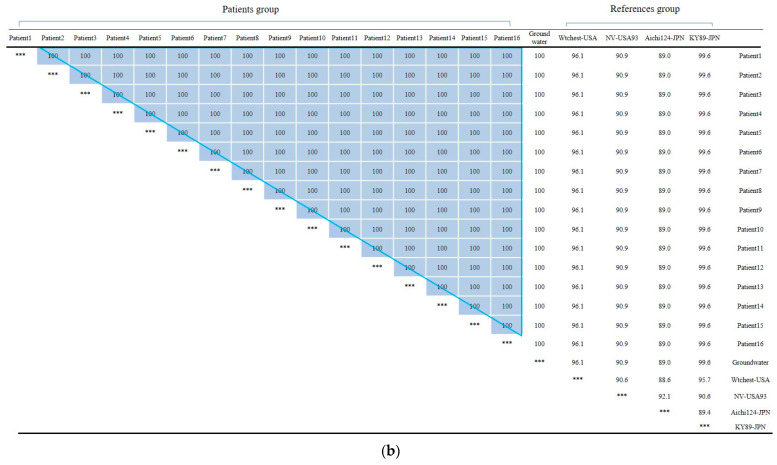
(**a**) Phylogenetic tree showing clustering of Norovirus strains isolated in food poisoning outbreak in Chungcheongnam-do, Republic of Korea: GI.1 and GI.8 strains of the Norovirus from Outbreak 3. (**b**) Nucleotide sequences showing GI.1 strains of the Norovirus from Outbreak 3. *** It meant that the variables on the X-axis and the Y-axis were the same. Blue big triangle meant patients clustering of the Norovirus.

Water and environmental samples were tested: 4 samples from groundwater; 4 samples from cooking utensils, such as knives and cutting boards; 9 samples from the living environment, such as bathroom doorknobs; 16 samples in total, including drinking water. As a result, out of 16, samples EAEC was detected in 1 sample and Norovirus was detected in 5 samples (Table 1). The specific genotypes of patients and environmental samples were GI.1 type in 16 patients and GI.8 type in 1 patient, and among five environmental samples, 3 were GI.1 type and 2 were GI.8 type (Figure 5a).

However, PFGE could not be conducted to analyze the relationship between EAEC detected in patients and cooking workers because EAEC is excluded from the Korea Centers for Disease Control and Prevention’s waterborne and foodborne management guidelines (KCDC, 2018).

In Outbreak 3, the GI.1-type Noroviruses isolated from 16 patients, 1 groundwater, 1 cafeteria cooking water, and 1 playground drinking water were 100% identical in the capsid region gene sequence of 256 bp. The GI.8-type Norovirus isolated from one floor of drinking water and one case of playground drinking water also had identical genes. The GI.1-type Norovirus group and the GI.8-type Norovirus group showed 74.6 to 77.3% homology, and the GI.1 type group showed 86.7 to 100% homology. Within the GI.8 type group, homology ranged from 94.5% to 100%.

## 3. Discussion

These outbreak data demonstrate that PFGE diagnosis and nucleotide sequencing method are instrumental in determining the cause of food poisoning. In the first epidemic case, four PFGE types of *S. aureus* were identified. Among these, STAS16.006 type was found in the rice cake and fish cake of the patient and the cook, making it possible to clearly determine the relationship between food poisoning pathogens. Previous research [13] reported that pulsed-field gel electrophoresis (PFGE), multilocus sequence typing (MLST), and spa typing methods were used to characterize *S. aureus* isolates from food surveillance from 2013–2015 in southwest China for *S. aureus* in southern China. From food surveillance, 163 PFGE-SmaI patterns were obtained for all the *S. aureus* samples. Among them, ST6-t701 (13.15%) was the most prevalent genotype. Additionally, the food from which *S. aureus* was most commonly isolated was grain products (56%) [13]. These findings from China are similar to the cause of the food poisoning epidemic caused by *S. aureus* in our study. The similarity in the causes of *S. aureus* food poisoning epidemics caused by grains is that, like in China, food poisoning epidemics caused by *S. aureus* in foods made from rice can occur in Korea, where rice and grains are the staple food.

In addition, the PFGE using PCR used in this study had the utility of screening pathogens that cause food poisoning with high sensitivity and specificity, as mentioned in previous studies [10,12]. Additionally, as shown in the results of this study, it can be used to detect multiple microbial groups and associations between causes of food poisoning and cooking employees [10]. For example, in the PFGE results of this study, STAS16.006 was found in common among four patients, one cooking employee, and food samples, making it possible to estimate the relationship between cooking employees and food poisoning. These results were like those of previous studies in China. The comparison between isolates from food and patients, where a high homology was observed for ST6-t701 types isolated from rice noodles and rice rolls, indicated the possible circulation of those types between patients and foods [13].

The fourth outbreak occurred after a group ate curry rice at a workplace because it was contaminated with EPEC, a crucial causative pathogen. Through PFGE testing, EPCX01.537 was found in both cooks, 17 patients, and preserved curry rice, excluding one human body, making it a case in which the cause of food poisoning could be identified. Other studies have reported that the occurrence rate of *E. coli* foodborne outbreaks has become higher over the past ten years. Foodborne outbreaks in that period have been chiefly caused by EPEC, STEC/EHEC, EIEC/Shigella, ETEC, and EAEC. In addition, most of these foodborne *E. coli* outbreak cases were attributed to the consumption of undercooked and contaminated food, such as ground beef hamburgers and uncooked vegetable salad [14,15]. EPEC was discovered in this study, and the cause in question was the curry rice, which was mixed with reservoir meat and vegetables but incompletely heated. This had something in common with the cause of *E. coli* food poisoning found in other studies [14]

The second and third food poisoning outbreaks occurred when drinking water was based on groundwater or when meals were prepared using groundwater. The pathogen investigation items and nucleotide sequencing procedures of the two outbreaks were almost identical. First, Norovirus, the most common cause of food poisoning, was identified as the causative agent [16,17,18]. While the virus was not detected in the preserved food, nor in all environmental samples related to groundwater, many of the patients and cooking employees were tested positive for Norovirus. Previous studies have identified contaminated groundwater as a cause of food poisoning [19,20]. The occurrence rate of Norovirus in groundwater has been reported to be approximately 8–21% worldwide [21]. In particular, numerous gastroenteritis outbreaks were caused by Norovirus contamination in drinking water [20].

The difference between the second and third Norovirus food poisoning outbreaks is that Norovirus was found in 12 cooking workers in the second outbreak while no Norovirus was found in cooking workers, and one case of *EAEC* was found in the third outbreak. In the case of the second outbreak, according to PFGE, the Norovirus genotypes were GI.7, GI.8, GII.2, GII.3, and GII.17, presenting a mixture of several types, while in the case of the third outbreak, only two genotypes—GI.1 and GI.8—were discovered. This difference in genotypes was due to the difference in the samples’ characteristics: the second food poisoning outbreak had a multi-sample of different ages and health conditions because they gathered from different areas to visit the winter festival. On the other hand, in the case of the third food poisoning outbreak, it was a single sample that used groundwater as drinking water in a high school. According to the previous research [22], the incidence rate of Norovirus depends on temperature. Outbreak 2 occurred in January, winter, and Outbreak 3 occurred in April, spring. Therefore, the differences in temperature could have affected the differences in the attack rates—37.6% and 27.3%, respectively—and subsequently in the genotypes [22].

Lastly, the detection rate of Norovirus was shown to have a seasonal and regional characteristic; only one sample isolated in winter was Norovirus-positive, while six out of seven were Norovirus-positive in summer. This may be because summer rainfall causes sewer overflows, making an environment that is easier for food poisoning outbreaks [19,20]. Therefore, GII accounts for most of the typical Norovirus epidemics [23]. Still, the frequency of reports of GI Norovirus food poisoning epidemics also depend on temperature, precipitation, and the spread of the epidemic [24,25,26,27]. In this study of Norovirus outbreak, in homology analysis with reference strains within the group of GI.1 type were the KY89-JPN(L23828) strain isolated in Japan in 1989 [28] and the Aichi124-JPN(AB031013) strain [29], which ranged from 89.0 to 99.6%, and the NV-USA93 (M87661) strain isolated in the United States in 1993 and 2001 [30] and the Wtchest-USA (AY502016) strain [31]. Therefore, it can be assumed that Norovirus can be widespread in a region due to seasonal and environmental factors as mentioned above as well as due to wild animals.

However, bacterial food poisoning testing through PFGE has some limitations. If the bacteria suspected of food poisoning are not identified or the suspect bacteria are not included in the test items and primers are not prepared to test them, the cause of bacterial food poisoning itself cannot be identified through PFGE. Additionally, depending on the measurement of the strain and the specificity of the results, there may be some inaccuracy in interpreting the results [32]. Among others, the biggest problem is that it is difficult to identify the infection route with the epidemiological investigation on its own even when systematic associations are found between a food poisoning epidemic and cooking employees. For example, whether the cooking employees is the direct cause or just a medium carrying the pathogen by touching or eating contaminated food. To overcome this problem, it is essential to obtain cooking employees’ medical histories, such as hospital visits or use of medication related to food poisoning symptoms before food poisoning outbreak. For example, if a food employee has a medical history of food poisoning treatment and a PFGE identifies a relation between the worker and the outbreak, it can be confirmed that the employee is the infection source. In addition, accurate identification of suspected patients through periodic and rapid monitoring and epidemiological investigation of bacterial food poisoning is also a prerequisite for more objective identification of food poisoning test results for PFGE [33].

The limitations of this study are as follows. This study administratively conducted PCR-based PFGE for bacteria and nucleotide sequencing for viruses on patient and environmental samples collected by public health centers that received reports of food poisoning in Chungcheong-do, Republic of Korea. Therefore, we could not perform PFGE analysis for *C. perfringens* and *EAEC*, which were not included in our administrative guidelines. Due to this limitation, additional research on these pathogens is also needed [34,35,36]. Second, this study is laboratory-based. If epidemiological investigators did not directly consider the relationship between the food poisoning outbreak and cooking employees, epidemiological information on the presence of food poisoning symptoms and hospital use history of cooking employees before the food poisoning outbreak could not be obtained. Therefore, more precise systems that combine pathogen test results with epidemiological information about the corresponding food poisoning outbreak are needed.

## 4. Materials and Methods

### 4.1. Outbreak Characteristics (Subjects)

Four food poisoning cases in the Chungcheongnam-do region between 2018 and 2020 were analyzed to identify food poisoning pathogens detected in cooking employees during food poisoning outbreaks. In order to identify the cause of food poisoning that occurred in groups, cases of food poisoning bacteria and viruses were tested in cases where people with symptoms such as vomiting, diarrhea, nausea, and abdominal pain were classified as patients. In addition, related food workers, cooking utensils, and environmental samples were examined. The four food poisonings analyzed in this study were outbreaks in four different regions (Table 1).

Based on the Infectious Disease Control and Prevention Act of the Republic of Korea, epidemiological investigators or infectious disease managers collected the test samples from the subjects as part of an administrative procedure to prevent and manage the spread of food poisoning. This study only used the anonymous laboratory results for the purpose of retrospective analysis to identify the pathogens and the relevance of cooking staff in the events of food poisoning. This study received an exemption for subject consent from an Institutional Review Board.

Outbreak 1 occurred at the OO Welfare Center in Boryeong-si, Chungcheongnam-do, with food poisoning bacteria and viruses were tested on 60 people (30 patients and 30 cooking employees) in Outbreak 1. *S. aureus* was detected in 16 of 30 patients and 1 of 30 cooking employees in the bacteria test, and no virus was detected. Additional tests were conducted with 19 commonly consumed preserved foods and 9 environmental samples, such as knives, cutting boards, dish towels, food streamers, drinking water, cooking water, and bottled water from the event places. As a result, out of 10 preserved foods, *S. aureus* was detected in 7 samples, including those of fish pancakes and rice cakes, and the same bacteria was detected in the samples of both patients and cooking employees. Neither bacteria nor viruses were detected in the nine environmental samples (Table 1). Outbreak 2 occurred at the OO Winter Festival in Cheongyang-gun, Chungcheongnam-do, with 38 patients, 32 cooking employees, and 7 environmental samples, including knives, cutting boards, and ground water. Outbreak 3 occurred at OO High School in Geumsan-gun, Chungcheongnam-do, with 38 patients, six cooking employees, 72 preserved foods, 95 environmental samples, knives, cutting boards, dishcloths, drinking water from the dormitory and cafeteria, doorknobs and basins in toilet, groundwater wells, hot and cold-water dispensers, cafeteria kitchen, school playground sinks. Outbreak 4 occurred at the OO workplace in Asan-si, Chungcheongnam-do, with 94 patients, 16 cooking employees, 135 preserved foods, and 12 environmental samples, such as knives, cutting boards, dish towels, and cooking water, were tested.

### 4.2. Sample Collection and Isolation of Pathogen

Preprocessing and testing of samples complied with the waterborne and foodborne infectious disease management guidelines (Korea Disease Control and Prevention Agency (KCDC), 2018), infectious disease laboratory diagnostic tests (KCDC, 2019), and food poisoning cause investigation guidelines (MFDS, 2018). To collect human samples, two or more swabs were collected from the rectum and mixed well in 3 mL of sterilized 0.1 M PBS (phosphate buffered saline, Sigma, St Louis, MO, USA). The samples were collected aseptically in sterilized containers at public health centers in the South Chungcheong region and were transported to the Chungcheongnam-do Health and Environment Research Institute for testing and analysis as quickly as possible while maintaining a low temperature of 4 °C without separately considering aerobic and anaerobic pathogens.

The samples were centrifuged at 3000 RPM for 10 min, and the supernatant was used. The environmental samples from kitchen utensils, such as doorknobs and cutting boards, were collected by swabbing widely using a sterilized cotton swab, and at least two samples were collected. For preserved food, the maximum amount was placed in a sterilized bag, diluted 10 times with saline solution, and shaken for bacterial culture. One liter of drinking water was filtered using a 0.45 μm membrane filter (Whatman, Tokyo, Japan) in a sterilized filtration device, and the filter paper was used for bacterial culture.

To detect viruses in water such as groundwater, a Nano Ceram filter (VS2.5-5, Argonide Corporation, Sanford, FL, USA) was attached to the Liquid Filtration System (1MDS-2000, 3M, Seoul, Republic of Korea) and filtered out 1500 L of groundwater to adsorb the virus [37]. The adsorbed virus was filtered by passing sterilized 1.5% beef extract buffer through a filter, and the filtered solution was slowly stirred at room temperature for 30 min at pH 3.5 ± 0.1. When a precipitate formed, it was centrifuged at 2500× *g*. The suspended solution was centrifuged again at 7000× *g* at 4 °C for 10 min, and the pH was adjusted to 7.0 ± 0.2. A 0.22 μm sterilized filter was attached to a 50 mL syringe to filter the supernatant to prevent bacterial contamination, and then nucleic acids were extracted using 20 mL of concentrated sample. The extracted nucleic acids were stored at −70 °C before testing.

The causative pathogens of food poisoning outbreaks analyzed in this study are presented in Table 2.

### 4.3. Genetic Fingerprint Analysis of Bacterial Testing in Food Poisoning

Testing and identification of food poisoning bacteria was conducted to find causative pathogens according to the Waterborne and Foodborne Infectious Disease Management Guidelines (KCDC, 2018) and the Food Poisoning Cause Investigation Test Method (MFDS, 2018), and human samples were tested according to the Infectious Disease Laboratory Diagnostic Test Method (KCDC, 2019). The environmental samples were conducted according to the food poisoning bacteria test method in Food Code IV.

The pre-treated specimen was added to Tryptic Soy Broth (Oxoid, Basingstoke, England), enriched, and cultured at 35 °C for 24 h, and then DNA (Deoxyribonucleic acid) was extracted. One mL of culture medium was taken and centrifuged at 8000 RPM for 1 min, the supernatant was removed, and 1 mL of PBS (Sigma, St Louis, MO, USA) was added and mixed well. After repeating this process three times, 500 μL of sterilized distilled water was added and heated at 100 °C for 15 min. Afterward, it was centrifuged at 8000 RPM for 30 s, the supernatant was used as template DNA, and specific gene amplification of each bacterium was performed. The bacteria causing food poisoning were tested using the PowerCheckTM 20 Pathogen Multiplex Real-time PCR Kit (Kogene Biotech, Seoul, Republic of Korea). Then, 5 μL of DNA was put into the kit, and it was mounted on ABI 7500 Fast real-time PCR (Thermo Fisher Scientific, Cleveland, OH, USA). After the initial reaction at 95 °C for 10 min, this procedure was repeated 40 times at 95 °C for 15 s and at 55 °C for 30 s. Genes for each bacterium were amplified in real time. The amplified gene was plotted in an exponential log graph, and a positive result was determined to be below 35 Ct (threshold cycle), the detection limit suggested by the kit. Bacteria tested positive were identified by isolating a single colony using the selective medium presented in IV—food poisoning bacteria test method and then conducting a biochemical test.

Pulsed-field gel electrophoresis (PFGE) was performed to analyze the genetic fingerprint of the isolated strain, and the analysis was submitted to the Bacteriological Analysis Department of the Korea Disease Control and Prevention Agency (KCDC, 2008). The restriction enzyme used for PFGE gene fingerprint analysis of *Staphylococcus aureus* was Sma I, and for Pathogenic *Escherichia coli*, Xba I was used [38].

### 4.4. Genotype Analysis of Virus Testing in Food Poisoning

Tests on pre-processed human and groundwater samples were performed according to Food Poisoning Virus Test Method V. of the Food Code. Nucleic acids were extracted using the QIAGEN RNA mini Kit (Qiagen, Hilden, Germany) and nucleic acid extraction equipment (Nextractor, Genolution, Seoul, Republic of Korea) according to the manufacturer’s experimental method. Nucleic acid was diagnosed using the PowerChekTM Norovirus GI/GII Multiplex Real-time PCR kit, PowerChekTM Sapovirus/Astrovirus Multiplex Real-time PCR Kit, PowerChekTM Adeno/Astro/Rotavirus Multiplex Real-time PCR Kit, PowerChekTM Hepatitis E Virus Real-time PCR Kit, and PowerChekTM Hepatitis A Virus Real-time PCR Kit (Kogene Biotech, Seoul, Republic of Korea). The extracted nucleic acids were measured for concentration and purity using QIAxpert (Qiagen, Hilden, Germany), and then 5 μL was added to each kit. In particular, each nucleic acid extracted from groundwater was performed three times in duplicate. After mounting on the ABI 7500 Fast real-time PCR (Thermo Fisher Scientific, Cleveland, OH, USA), the reaction conditions were 50 °C once for 30 min, 95 °C once for 10 min, 95 °C for 15 s, and 55 °C for 1 min 45 times. The standard for judging positivity was a threshold of 0.2 and a Ct value 36.

For the Norovirus that was determined to be positive, the capsid gene was amplified by semi-nested RT-PCR to analyze the genotype. The extracted RNA was tested using the Access RT-PCR system (Promega, Madison, WA, USA), including primers specifically for Norovirus. Norovirus GI primers are GI-F1M (5′-CTGCCCGAATTYGTAAATGATGAT-3′) and GI-R1M (5′-CCAACCCARCCATTRTACATYTG-3′), and Norovirus GII primers GII-F1M (5′-GGGAGGGCGATCGCAATCT-3′) and GII-R1M (5′-CCRCCIGCATRICCRTTRTACAT-3′) were used. After adding 5 μL of RNA (ribonucleic acid) to each kit, they were synthesized with cDNA (complementary DNA) at 47 °C for 40 min, repeating 35 times 30 s at 94 °C, 30 s at 54 °C, and 45 s at 72 °C. A PCR reaction was performed. Afterward, semi-nested PCR was performed using a 2 μL PCR system of the first reaction product (Promega, Madison, WA, USA). Norovirus GI primers were GI-F2 (5′-ATGATGGCGTCTAAGGACGC-3′) and GI-R1M, and GII primers were GII-F3 (5′-TTGTGAATGAAGATGGGCGTCGART-3′) and GII-R1M. The reaction conditions were 94 °C for 30 s, 56 °C for 30 s, and 72 °C for 45 s, repeated 25 times. For the Norovirus that was determined to be positive, the capsid gene was amplified by semi-nested RT-PCR to analyze the genotype. After the extracted RNA was quantified by using the QIAxpert system (Qiagen, Hilden, Germany), it was tested using the Access RT-PCR system (Promega, Madison, WA, USA), and a band of 330 bp for GI and 332 bp for GII was confirmed.

The PCR product was commissioned from Bioneer (Bioneer, Daejeon, Republic of Korea), had its base sequence analyzed, and was registered in ENTERNET, part of the Korea Centers for Disease Control and Prevention’s integrated disease and health management system. Each nucleotide sequence was edited in Editseq (DNASTAR, Madison, WI, USA), and the genotype was determined by comparison with the nearest original strain using the basic local alignment search tool (BLAST) of the National Center for Biotechnology Information. Nucleotide sequences were aligned with each genotype’s isolated Norovirus and reference strains after multiple alignments using the Clustal W method [39] implemented in MegAlign (DNASTAR, Madison, WI, USA). Phylogenetic analyses were performed using MEGA version 6.0 software [40].

## 5. Conclusions

PCR-based PFGE of bacterial food poisoning and Genotype analysis of virus testing presented the highest prevalence of the Norovirus. In addition, it helped identify the cause of a food poisoning epidemic and its relationship with cooking employees. However, it was difficult to determine whether cooking employees were the direct cause of the food poisoning epidemic based on these molecular biological diagnostic results alone. Consequently, a more effective system needs to be developed to integrate epidemiological and diagnostic information to identify a direct correlation between the food poisoning epidemic and cooking employees.

## Figures and Tables

**Table 1:** Outbreak characteristics of food poisoning in Chungcheongnam-do, Republic of Korea.

Outbreak	Outbreak 1	Outbreak 2	Outbreak 3	Outbreak 4
Date of occurrence	30 November 2018	9 January 2019	11 April 2019	25 June 2020
Place of occurrence	Boryeong	Chenongyang	Geumsan	Asan
Facility of outbreak	Welfare center	Winter festival	High school	Cafeteria in company
Attack rate (%)	26/200 (13%)	38/101 (37.6%)	53/194 (27.3%)	94/402 (23.4%)
Incubation Period (mean hour)	1–6.2 (4.7)	33–41		23.5–40.5 (31.2)
Suspected food & water	Rice cake, Fish pancake	Underground water	Underground water	Curry rice
Pathogen	*S. aureus*	Norovirus	Norovirus EAEC *S. aureus*	EPEC *C. perfringens*
Method	PFGE	Nucleotide sequencing	Nucleotide sequencing	PFGE
No. of positive/tested (%)				
Patient	16/30 (53.3%)	22/29 (75.9%)	17/39 (43.6%) 21/39 (53.8%) 7/39 (17.9%)	12/82 (14.6%) 13/82 (15.8%)
Cooking employee	1/30 (3.3%)	12/32 (37.5%)	0/6 (0%) 1/6 (16.7%) 0/6 (0%)	3/16 (18.8%) 0/16 (0%)
Preserved food	7/10 (70.0%)	0/0 (0%)	0/72 (0%) 0/72 (0%) 0/72 (0%)	1/135(0.7%) 0/135 (0%)
Environment Sample *	0/9 (0)	2/7 (28.6)	5/19 (26.3) 1/95 (1.1%) 0/95 (0%)	0/12 (0%) 0/12 (0%)
Cooking employees’ possibility of being the source of infection	Possible	Unlikely **	Unlikely ** Unknown Unknown	Possible Unlikely

* Environment samples include groundwater, groundwater wells, hot- and cold-water dispensers, drinking water in the kitchen, groundwater used in school playgrounds, knives, cutting boards, dishcloths, and cooking utensils. ** In Outbreaks 2 and 3, it was more reasonable to assume that the cooking workers were exposed to groundwater contaminated with Norovirus, which causes food poisoning.

**Table 2:** The causative pathogens of food poisoning cases analyzed in this study.

	Pathogens
Bacteria	*Salmonella* spp., *Staphylococcus aureus*, *Vibrio parahaemolyticus*, *Vibrio cholerae*, *Vibrio vulnificus*, *Listeria monocytogenes*, *Shigella* spp., *Bacillus cereus*, *Yersinia enterocolitica*, *Campylobacter jejuni*, *Campylobacter coli*, *Clostridium perfringens*, *Clostridium botulinum*, Pathogenic *Escherichia coli* (Enteropathgenic *E. coli* (EPEC), Enterohaemorrhagic *E. coli* (*EHEC*), Enterotoxigenic *E. coli* (*ETEC*), Enteroaggregative *E. coli* (*EAEC*), Enteroinvasive *E coli (EIEC*))
Viruses	Norovirus, Hepatitis A virus, Rotavirus, Astrovirus, Sapovirus, Enteric Adenovirus, Hepatitis E virus

## Data Availability

All data are available from the corresponding author upon reasonable request.
